# The regional myocardial infarction registry of Saxony-Anhalt (RHESA) in Germany – rational and study protocol

**DOI:** 10.1186/s12872-015-0040-2

**Published:** 2015-06-09

**Authors:** Stefanie Bohley, Pietro Trocchi, Bernt-Peter Robra, Wilfried Mau, Andreas Stang

**Affiliations:** Institute of Medical Epidemiology, Biostatistics and Informatics, Martin Luther University Halle-Wittenberg, Halle (Saale), Germany; Institute of Social Medicine and Health Economics, Otto-von-Guericke-University Magdeburg, Magdeburg, Germany; Institute of Rehabilitation Medicine, Martin Luther University Halle-Wittenberg, Halle (Saale), Germany; Center of Clinical Epidemiology, Institute of Medical Informatics, Biometry and Epidemiology, University Hospital of Essen, Essen, Germany; Department of Epidemiology, School of Public Health, Boston University, Boston, USA

**Keywords:** Myocardial infarction, Registry, Germany, Epidemiology, Cardiovascular disorders, RHESA

## Abstract

**Background:**

In 2012 the age-standardized acute myocardial infarction (AMI) mortality rate was in the federal state Saxony-Anhalt 67 deaths per 100.000 whereas in Germany the AMI-rate was 47 deaths per 100.000. The rate in Saxony-Anhalt was therefore 43 % above the national average. Many factors may explain this above-average AMI mortality rate:

First, the prevalence of cardiovascular risk factors (e.g. arterial hypertension, diabetes mellitus, smoking) in Saxony-Anhalt is the highest among all the Federal States of Germany. Second, structural health care for patients with AMI is potentially deficient (e.g. insufficient number of percutaneous coronary intervention-centers or deficits in the pre-hospital logistics of care). Third, the pre- and in-hospital process quality of health care for patients with AMI is possibly insufficient (e.g. time to reperfusion therapy).

In July 2013 we established the regional myocardial infarction registry of Saxony-Anhalt (Regionales Herzinfarktregister in Sachsen-Anhalt, RHESA). RHESA is a population-based registry in the eastern part of Germany.

Aims of RHESA are to calculate the AMI morbidity and mortality rates. Furthermore we study the factors that may potentially influence these rates in Saxony-Anhalt.

**Methods:**

RHESA is a population-based registry of patients with fatal or non-fatal AMI that was established in July 2013. The registry population comprises inhabitants aged 25 years or more of the city of Halle (Saale) (n = 179.000) and inhabitants of the rural district Altmark (n = 165.000) in the federal state Saxony-Anhalt, Germany.

**Discussion:**

The main objectives of RHESA are to provide detailed estimates of the burden of AMI in Saxony-Anhalt which is the federal state with the highest AMI mortality rate in Germany and to investigate factors that influence morbidity and mortality rates due to AMI. Data collected in RHESA enable us to assess different levels of quality of health care of patients with AMI (structural, process and outcome). RHESA provides for the first time estimates of the burden of AMI in Saxony-Anhalt, and therefore contributes considerably to an improvement of the German Health Monitoring that strives for a more valid extrapolation of the nationwide morbidity and mortality rates of AMI.

**Electronic supplementary material:**

The online version of this article (doi:10.1186/s12872-015-0040-2) contains supplementary material, which is available to authorized users.

## Background

Cardiovascular diseases (CVD) are the leading causes of death and morbidity in Europe. In 2012, CVD caused 47 % of all deaths in Europe [1] and 40 % in Germany [2]. For Germany a decrease in CVD mortality over the past decades has been observed. However, an east–west and north–south gradient of higher mortality in the eastern and northern part is still present. For the year 2012, the highest age-standardised death rates were observed in Saxony-Anhalt, Mecklenburg-Western Pomerania, and the lowest in Hamburg, Berlin, Baden-Wuerttemberg, and Hesse [2].

Differences in mortality rates due to acute myocardial infarction (AMI) across the federal states of Germany are also present. The age-standardised AMI mortality rate in 2012 was 67 deaths per 100.000 person-years in Saxony-Anhalt, 43 % above the national average of 47 deaths per 100.000 [3].

Many factors may explain the higher AMI mortality rate in Saxony-Anhalt. First, the prevalence of cardiovascular risk factors (diabetes mellitus, smoking, arterial hypertension, obesity, increased waist circumference, and metabolic syndrome) in Saxony-Anhalt is the highest among all Federal States of Germany [4]. Second, structural quality of health care for patients with AMI is potentially inadequate, e.g. less percutaneous coronary intervention-centers (PCI-centers), chest-pain units (CPU), and cardiologists compared to the national average (Table 1). Third, the pre- and in-hospital process quality of health care for patients with AMI could be less sufficient (e.g. patient delay, system delay, time to reperfusion therapy) [5].

**Table 1:** Number of chest pain units, PCI-centers, and cardiologists per 100.000 inhabitants in Saxony-Anhalt and Federal Republic of Germany [13]

	Saxony-Anhalt	Germany
Chest Pain Units	0,09	0,26
PCI Centers	0,94	1,06
Cardiologists	3,12	3,96

Currently, population-based event rates of AMI in Germany are based on data from the region of Augsburg, Bavaria (AMI Registry of the Cooperative Health Research of the Region Augsburg, KORA [6]). Furthermore there are some clinical registries like the Berlin Myocardial Infarction Registry, BMIR [7]. Alternatively, source of aggregate data for morbidity rates of AMI are from diagnosis-related groups statistics and health insurance funds, routinely collected data that are subject to selection bias.

The regional myocardial infarction registry of Saxony-Anhalt (Regionales Herzinfarktregister Sachsen-Anhalt, RHESA) is a population-based registry of fatal and non-fatal cases of myocardial infarction in this federal state of Germany.

Primary aims of RHESA are to calculate AMI morbidity and mortality rates in Saxony-Anhalt and to study the factors that may potentially influence these rates. Furthermore, by systematic follow-up of registered AMI patients, survival and determinants of survival are studied.

In particular RHESA enables us:

1. to assess quality of health care of AMI patients [8] in terms of
  structure (related to number of PCI Center, structure of emergency services [9])
  process (different time intervals: symptom onset to first medical contact (FMC), FMC to Diagnosis, FMC to reperfusion therapy, pain to door, door to balloon [DTB])
  outcome (pre-, −in-hospital AMI mortality, mortality of AMI survivors, re-infarction rate)
2. to compile a risk- and care-profile of the AMI-patients.
3. to assess regional disparities in AMI management (rural vs. urban region)

This paper presents the design of the Regional Myocardial Infarction Registry of Saxony-Anhalt (RHESA).

## Methods/Design

### Study design

RHESA is a population-based registry of patients with AMI that was established in July 2013 and is conducted by the Institute of Medical Epidemiology, Biostatistics and Informatics at the Martin-Luther-University Halle-Wittenberg, Germany.

### Study population

**Fig. 1:**
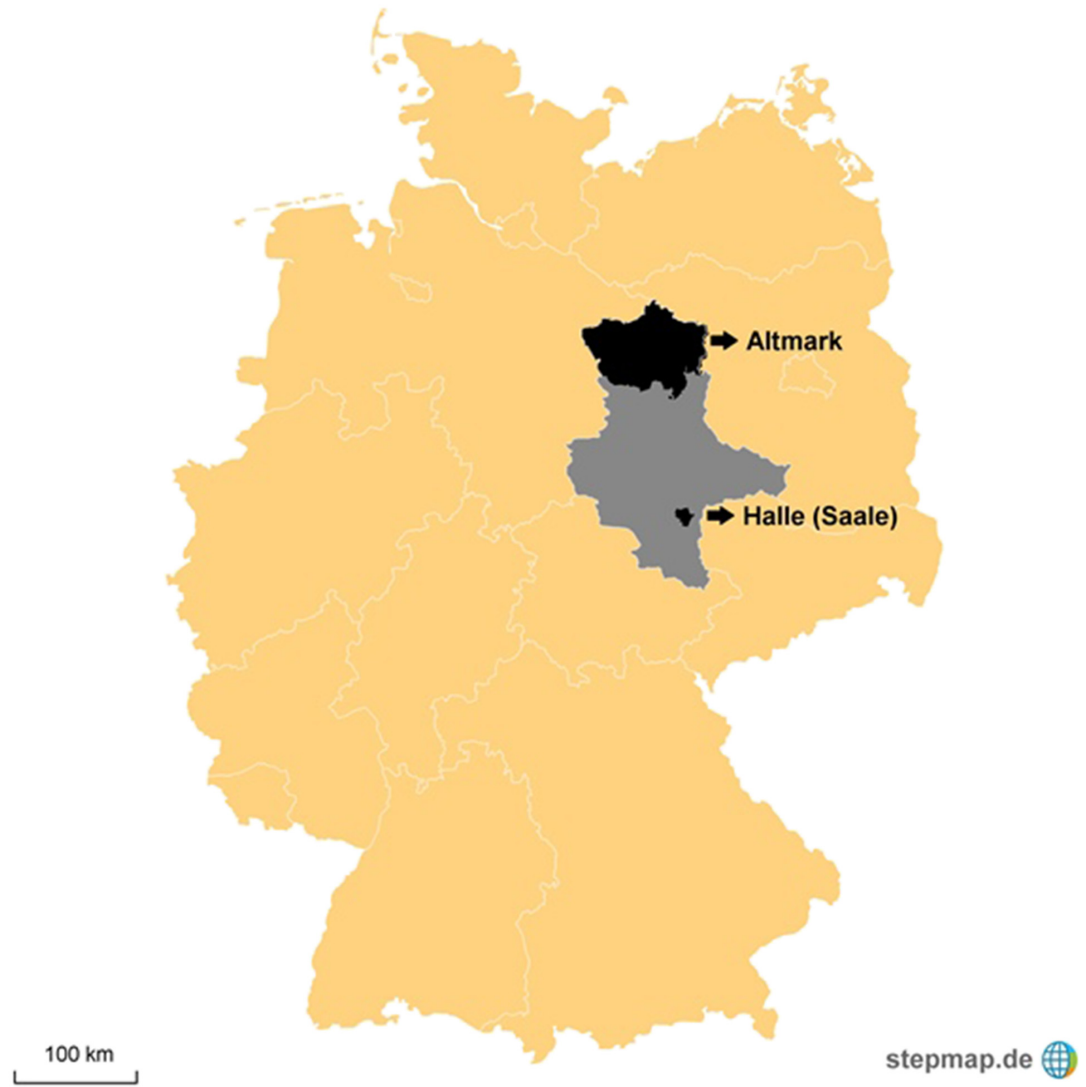
Region of Registry (Yellow = Germany, Grey = federal state Saxony-Anhalt, Black = registry region: City of Halle (Saale), Altmark)

The registry population comprises inhabitants aged 25 years or more of the city of Halle (Saale) (n = 179.000; population density: 1.326 per km^2^) and inhabitants of the rural district Altmark (n = 165.000, population density: 35 per km^2^) in the federal state Saxony-Anhalt, Germany (Fig. 1). Collaborating institutions in the region are: 16 hospitals, three health departments (Halle, Salzwedel, and Stendal), the rescue coordination centre in Halle (Saale), the rescue service “Johanniter” in Stendal, 16 residence registration offices, and approx. 550 physicians.

### Data collection

**Fig. 2:**
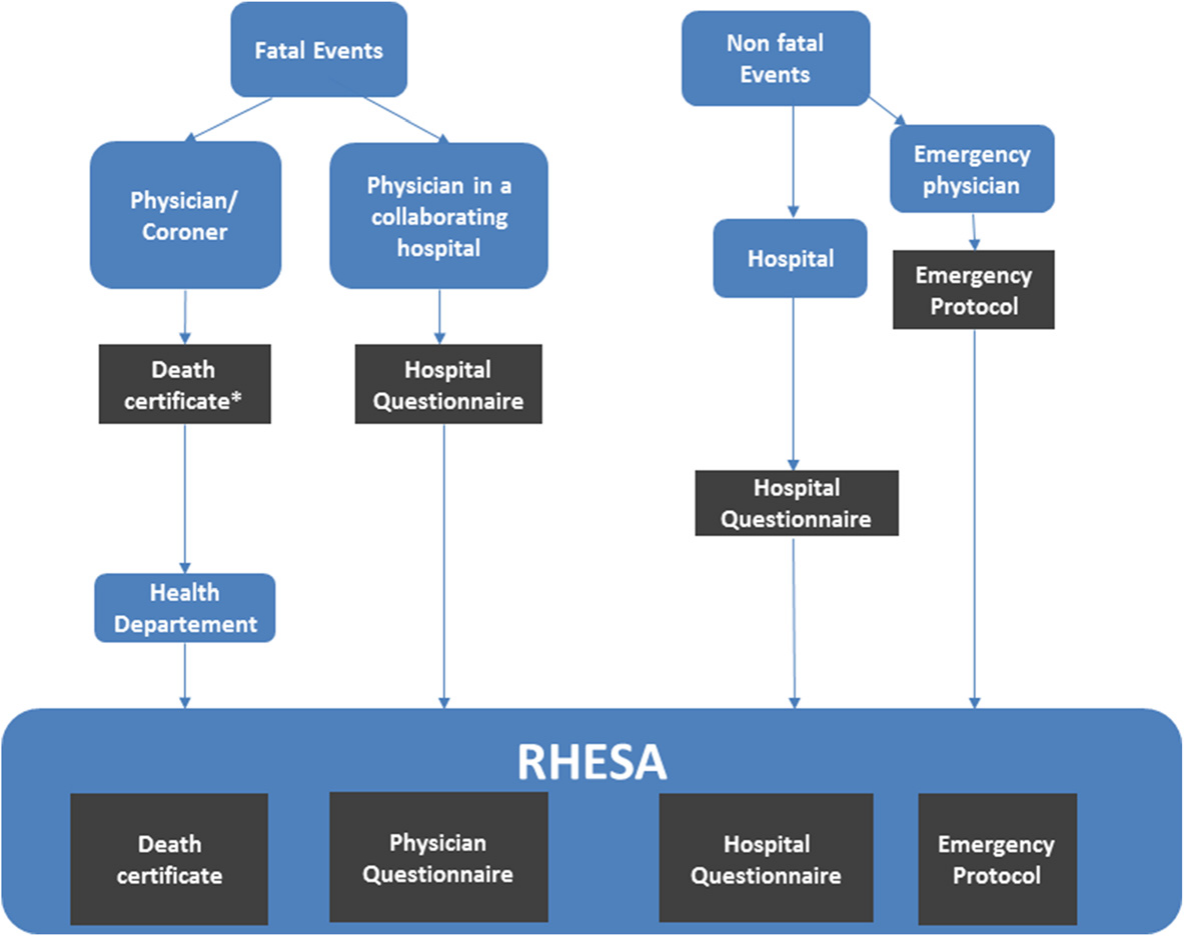
Schema of RHESA data collection. *completed with a Physician Questionnaire

Figure 2 shows the stepwise description of the RHESA data collection.

#### Non-fatal events

According to the “Third universal definition of myocardial infarction” of the European Society of Cardiology we define an AMI if there is evidence of myocardial necrosis in a clinical setting consistent with acute myocardial ischaemia. “Under these conditions is an acute myocardial infarction defined as: Detection of a rise and/or fall of cardiac biomarker values (preferably cardiac troponin [cTn]) with at least one value above the 99th percentile upper reference limit and with at least one of the following: symptoms of ischaemia, new or presumed new significant ST-segment–T wave changes or new left bundle branch block, development of pathological Q waves in the ECG, imaging evidence of new loss of viable myocardium or new regional wall motion abnormality, identification of an intracoronary thrombus by angiography or autopsy” [10].

All patients with a clinically confirmed AMI are asked to give written consent to participate in the registry of RHESA by the medical staff (physicians or study nurses) of the collaborating hospitals. Trained physicians or study nurses collect data with a hospital questionnaire during patients’ hospitalization by medical record review. Data include 1) personal data, 2) preexisting risk factors and comorbidities, 3) medical treatments and interventions during hospitalization, 5) clinical complications, 6) medication before and during hospitalization and at hospital discharge [see Additional file 1]. The questionnaire was derived from the BMIR [7]. From potentially eligible patients who refuse to participate, a hospital questionnaire with anonymised data is filled in.

From each patient who consented to participate in RHESA, an emergency protocol completed by an emergency physician (mostly in the ambulance) is also collected. Information from the emergency protocols allows investigations into emergency management in the prehospital phase. Items of interest are: 1) duration from symptom onset until emergency call, 2) duration until the arrival at the patient’s home, 3) duration of the medical treatment of the emergency physician on site, 4) duration until arrival at the hospital, 5) treatments and interventions, and 6) medication.

#### Fatal events

Fatal events are defined according to the registry of the WHO-MONICA (Multinational MONItoring of trends and determinants in CArdiovascular disease)/KORA study [11].

The three health departments send anonymised death certificates to RHESA monthly that contains at least one of the following diagnoses: hypertension (ICD-10 I10), ischaemic heart disease (ICD-10 I20-25), other CVD, including sudden cardiac death (ICD-10 I30-I52), atherosclerosis (ICD-10 I70), diabetes mellitus (ICD-10 E10-E11), dyslipidemia (ICD-10 I78), obesity (ICD-10 E66). Furthermore the health departments send a physician’s questionnaire [see Additional file 2] to the most recent treating physician or coroner. These physicians send the filled physician’s questionnaire back to RHESA. We use the information from the death certificate and the physician’s questionnaire for classifying events according to the MONICA diagnostic category of coronary death. We classify four categories: “definite acute myocardial infarction” (autopsy), “possible coronary death” (acute symptoms, and a positive history of ischaemic heart disease [angina pectoris or previous myocardial infarction or diagnosed ischaemic heart disease]), “no acute myocardial infarction” (where another diagnosis has been made (clinically or at autopsy), or “fatal cases with insufficient data” (cases with no autopsy, no history of typical or atypical or inadequately described symptoms, no previous history of ischaemic heart disease and no other diagnosis) [12]. Details of definitions of the variables are given in the respective design papers [12, 11].

For the study of the survival status among AMI patients who gave consent for follow-up and re-contact, the registration offices are contacted at different points in time. For patients who have died, the responsible health department sends the corresponding death certificate to RHESA.

### Follow up

Since November 2014, we have been conducting telephone interviews with participating AMI patients (RHESA-CARE-Study). We collect data about cardiovascular risk factors, medication and utilization of medical services before and after the AMI, psychosocial factors (socioeconomic and employment status, depression), and participation in cardiovascular rehabilitation.

### Quality assurance

We established different methods for quality assurance.

1. Standard Operating Procedures
  To reduce variability in data collection, all reporting institutions (hospitals, health departments) fill in all questionnaires according to standard operating procedures.
2. Data-Completeness
  All records are regularly checked for errors and inconsistencies.
3. Case-Reporting-Completeness
  To check the completeness of case-reporting, we compare the number of all patients who were annually discharged from our 16 collaborating hospitals with diagnosis of AMI (ICD-10: I20, I21) with the number of registered cases.

- Twice a month, every reporting hospital receives a RHESA reminder with the slogan: “Already reported?”
- Once a month, every reporting hospital receives a diagram with the number of incoming case reports
- Twice a year, the participating centres receive a detailed report from RHESA. The report comprises data on diagnostics, treatments, medications, and time intervals (e.g. DTB). Furthermore hospitals can compare their own results with those from other centres.
- Once a year, we organize a conference with all reporting hospitals.

### Statistical analyses

We calculate several epidemiologic frequency measures of interest. First, we calculate age- and sex-specific AMI morbidity rates (per 100.000 person-years) by dividing the number of incident and recurrent cases of AMI by the mid-year population of Saxony-Anhalt in the respective calendar year. Second, we calculate age- and sex-specific AMI mortality rates (per 100.000 person-years) by dividing the total number of cases of AMI followed by death by the mid-year population of Saxony-Anhalt in the respective calendar year. Third, we calculate age- and sex-specific case fatality rates (in %) by dividing the number of cases of AMI followed by death within a defined time interval of onset (before being admitted to hospital, within 24 h and within 28 days) by the total number of cases of AMI. Statistical analyses are performed using SAS 9.4 (SAS Institute, Cary, NC, USA).

### Ethics

RHESA was approved by the Ethics Committee of the Medical Faculty of the Martin-Luther-University Halle-Wittenberg and by the State Data Protection and Privacy Commissioner of Saxony-Anhalt.

## Discussion

The main objectives of RHESA are to provide detailed estimates of the burden of AMI in Saxony-Anhalt and to investigate factors that influence morbidity and mortality rates due to AMI. Data collected in RHESA enable us to assess different levels of quality of health care of patients with AMI (structural, process and outcome).

In particular, data on event rates of AMI have important implications for the allocation of funds for the improvement of the effectiveness of management and health care provision of patient who suffered from AMI (with diagnosis of AMI) and the strengthening of epidemiological research in the area of the health care services.

Finally, for the first time RHESA provides estimates of the burden of AMI in Saxony-Anhalt, which is the federal state with the highest AMI mortality rate in Germany, and therefore RHESA contributes considerably to an improvement of the German Health Monitoring that strives for a more valid extrapolation of the nationwide morbidity and mortality rates of AMI.

## Additional files

Additional file 1:
**Hospital questionnaire.**
Additional file 2:
**Physician’s questionnaire.**

## Abbreviations

AMI: Acute myocardial infarction
BMIR: Berlin Myocardial Infarction Registry
CHD: Coronary heart disease
CVD: Cardiovascular diseases
DTB: Door to balloon
FMC: First medical contact
KORA: Cooperative Health Research of the Region Augsburg
PCI: Percutaneous coronary intervention
RHESA: Regional Myocardial Infarction Registry of Saxony-Anhalt
WHO-MONICA: World Health Organization-Multinational MONItoring of trends and determinants in CArdiovascular disease
