# Supplementary material for: The regional myocardial infarction registry of Saxony-Anhalt (RHESA) in Germany – rational and study protocol
Source: BMC Cardiovasc Disord. 2015 Jun 9;15:45. doi: 10.1186/s12872-015-0040-2 (PMC4467162; doi:10.1186/s12872-015-0040-2)
Supplement: Additional file 1: — Hospital questionnaire. [file 12872_2015_40_MOESM1_ESM.pdf]

Hospital Questionnaire (Version 4.0)

Regional Myocardial Infarction Registry Saxony-Anhalt

Hospital-Code ☐

Martin-Luther-University Halle-Wittenberg
Medical Faculty
IMEBI
06097 Halle (Saale)

Tel.: 0345 557-3579
Fax: 0345 557-3565
rhesa@medizin.uni-halle.de
www.medizin.uni-halle.de/rhesa/de

Inclusion criteria
Confirmed diagnosis myocardial infarct (STEMI and NSTEMI) | **A1** | **consent is given**

1. Personal data

Name ____________________

First name ____________________

Birthdate |__||__|.|__||__|.|__||__||__||__| (Day Month Year) ☐ Man ☐ Woman

Weight ________ kg Height ________ cm

____
Postcode, place of residence

____________________ ____________________
Name of family physician health insurance

____
Assignment number from emergency protocol

2. First care

Symptom-onset |__||__|.|__||__|.||__||__| |__||__|.|__||__| ☐ n. k.
(Day Month Year Hour Min.)

Hospital admission|__||__|.|__||__|.||__||__| |__||__|.|__||__| ☐ n. k.
(Day Month Year Hour Min.)

Intrahospital ☐ yes ☐ no ☐ n.k.

First treatment ☐ NEF ☐ RTW ☐ RTH/ITH ☐ KTW ☐ KV-Dienst ☐ alone ☐ n.k.

First aid (lay) ☐ yes ☐ no ☐ n.k.

Assignment ☐ ja ☐ nein ☐ n.k.

If yes, from which: ____________________

3. Risk factors and comorbidities

Smoker	☐ yes	☐ formerly	☐ no	☐ n.k.
Diabetes mellitus	☐ yes	☐ new	☐ no	☐ n.k.
Hypertension	☐ yes		☐ no	☐ n.k.
Hypercholesterolemia	☐ yes		☐ no	☐ n.k.
Post-myocardial infarction status	☐ yes		☐ no	☐ n.k.
Post-PCI status	☐ yes		☐ no	☐ n.k.
Post-Bypass-OP status	☐ yes		☐ no	☐ n.k.
Post-Apoplexy status	☐ yes		☐ no	☐ n.k.
Atrial fibrillation/flutter	☐ yes		☐ no	☐ n.k.
Heart failure	☐ yes		☐ no	☐ n.k.
Renal failure	☐ yes		☐ no	☐ n.k.
pAVK	☐ yes		☐ no	☐ n.k.

4. Acute diagnostic investigations

On admission HF |__||__||__|/Min.

RR |__||__||__|/|__||__||__| mmHg

Cardiogenic shock on admission:
☐ yes ☐ no ☐ n.k.

First EKG ☐ STEMI ☐ NSTEMI ☐ new LSB

☐ Troponin I ☐ Troponin T Value |________| Threshold value___|

☐ Type 1 ☐ Type 2 ☐ Type3 ☐ Type4a ☐ Type4b ☐ Type5 ☐ n.k.

First attending station:
☐ ITS ☐ IMC/CPU
☐ Station with monitoring system ☐ Normalstation

5. First therapy (PCI see 5a)

☐ ASS ☐ Clopidogrel/Prasugrel/Ticagrelor ☐ Heparin
☐ GP IIb/IIIa-Antagonist

Thrombolytic therapy ☐ ja |__||__|.|__||__|.||__||__| |__||__|.|__||__|
(Day Month Year Hour Min.)
☐ no ☐ n. k.

If yes, which substance: ____________________ ☐ n. k.

Bypass-OP ☐ yes ☐ no ☐ n. k.
If yes, ☐ elective or
☐ Emergency-OP: |__||__|.|__||__|.|__||__|
(Day Month Year)

Why neither thrombolytic therapy nor PCI nor CABG:
____

5a. PCI

PCI ☐ yes ☐ no
If yes, access: ☐ transradial ☐ transfemoral
Stent ☐ yes ☐ no
If yes, ☐ BMS ☐ DES

Date PCI: |__||__|.|__||__|.|__||__|
(Day Month Year)

Vessel puncture |__||__|.|__||__| First Ballon |__||__|.|__||__|
(Hour Min. Hour Min.)

TIMI (before) __________ TIMI (afterwards) __________
IABP ☐ yes ☐ no

6. Stationary complications

☐ none
☐ Shock (new) ☐ Intubation ☐ Resuscitation
☐ Re-infarct ☐ Stroke ☐ Re-intervention
☐ Severe bleeding

☐ Other: ____________________

7. Medications at discharge

☐ ASS ☐ Beta blocker ☐ ACE/ARB-Hemmer
☐ Clopidogrel/Prasugrel/Ticagrelor
☐ Diuretic ☐ Anticoagulant ☐ Aldosteronantagonist
☐ Statine ☐ other Cholesterol lowering drug
☐ Insulin ☐ oral Antidiabetic drug

☐ Other: ____________________

8. Discharge, Transfer, Death

☐ Patient discharged Date: |__||__|.|__||__|.|__||__|
(Day Month Year)

☐ Patient transferred Date: |__||__|.|__||__|.|__||__| |__||__|.|__||__|
(Day Month Year Hour Min.)

If Patient transferred, transfer to:
☐ Bad Bevensen ☐ Magdeburg ☐ Perleberg
☐ Stendal ☐ Uelzen ☐ Wittstock

☐ Other: ____________________

☐ Patient died Date: |__||__|.|__||__|.|__||__| |__||__|.|__||__|
(Day Month Year Hour Min.)

Questionnaire filled: |__||__|.|__||__|.|__||__|
(Day Month Year)

____
Name oft he Physician (block capital)

____
Signature oft he physician
