# Supplementary material for: The regional myocardial infarction registry of Saxony-Anhalt (RHESA) in Germany – rational and study protocol
Source: BMC Cardiovasc Disord. 2015 Jun 9;15:45. doi: 10.1186/s12872-015-0040-2 (PMC4467162; doi:10.1186/s12872-015-0040-2)
Supplement: Additional file 2: — Physician’s questionnaire. [file 12872_2015_40_MOESM2_ESM.pdf]

Regional Myocardial Infarction Registry Saxony-Anhalt (RHESA)

Stamp of the doctor's office

Datum Signature

Account holder

IBAN

BIC

Bank

☐ I waive the payment of allowances in favor of RHESA.

Information about the family doctor or last attending physician (if known)

Name

Address

Information from the death certificate (to be completed by the health department

Year of certification	|__|__||__|__|
Municipal code - registry office:	|__|__||__|__||__|__||__|__|
Death registry-Nr.:	|__|__||__|__||__|__||__|__|

In the determination of death by the emergency service please use the number of the emergency protocol:

____

PHYSICIAN'S - QUESTIONNAIRE

1. Were you: ☐ Family doctor or last treating physician ☐ autopsy physician

Information about the deceased person:

2. Was the deceased living alone?
☐ Yes ☐ No ☐ Not known

3. Occupational situation of deceased before death
☐ Employed ☐ Not employed ☐ Not known

Questions about dead circumstances:

4. *If the patient died in in-patient clinic:*
4.1. Where died the patient?

☐ Hospital admission
☐ General care unit ☐ Intensive care unit
☐ Rehabilitation centre ☐ Hospice

☐ Other: ______________________________________
☐ Not known

4.2. *If the death occurred in hospital:*
Was the last hospital admission primarily due to an acute myocardial infarction?

☐ Yes ☐ No

If no, which admission diagnosis:

____

____

5. Have you met the deceased yet alive?
☐ Yes ☐ No

6. Died the patient in presence of witnesses?
☐ Yes ☐ No ☐ Not known

7. If the rescue service called?
☐ Yes ☐ No ☐ Not known

8. If the resuscitation carried out?
☐ Yes ☐ No ☐ Not known

9. Where and by whom was the resuscitation carried out?
(multiple choice possible)

☐ Hospital admission
☐ Intensive care unit
☐ General care unit
☐ Other care unit in hospital
☐ Outside of the hospital
☐ by physician
☐ by ambulance officer or paramedic
☐ by other healthcare professionals
☐ by laymen

☐ Other: ______________________________________
☐ Not known

Questions about coronary event:

☐ Patient did not die because of a myocardial infarct
(If so, please continue from question 16)

10. How long was the time interval between the onset of acute pain due to heart attack and the occurrence of death?

Hours: _______________ Minutes:________________

☐ According to witnesses
☐ According to personal observation
☐ Not known

PHYSICIAN'S - QUESTIONNAIRE

Questions about coronary event:

11. Blamed the deceased in the last hours before death disorders that would indicate the presence of myocardial infarct?

- ☐ Yes, according to the patient, relatives or witnesses
- ☐ Yes, according to personal observation
- ☐ No ☐ Not known

12. Was a matter of strong, persistent, precordial pain?

☐ Yes ☐ No ☐ Not known

13. Was a thrombolitic therapy carried out?

☐ Yes, systemic ☐ Yes, intra-coronary ☐ No
☐ Not known

14. Was a PTCA carried out?

☐ Yes, with Stent ☐ Yes, without Stent ☐ No
☐ Not known

15. Was a Bypass-Operation carried out?

☐ Yes ☐ No ☐ Not known

Questions about anamnesis and long-term medications:

16. Had the deceased earlier (that is, more than 4 weeks before the death) a myocardial infarct, that was treated at the hospital?

☐ Yes ☐ No ☐ Not known

17. Had the deceased anamnestic evidence of angina pectoris?

☐ Yes ☐ No ☐ Not known

18. Had the deceased a confirmed clinical evidence of coronary heart disease?

☐ Yes ☐ No ☐ Not known

If yes, which investigations were carried out?

____

____

____

19. Was carried out in the patient earlier (that is, more than 4 weeks before the death) a bypass-surgery?

☐ Yes ☐ No ☐ Not known

20. Was carried out in the patient earlier (that is, more than 4 weeks before the death), a PTCA?

☐ Yes, with Stent ☐ Yes, without Stent ☐ No
☐ Not known

21. Was there anamnestic evidence of one of the following disease?
(multiple choice possible)

- ☐ Hyperlipidemia
- ☐ Hypertension
- ☐ Apoplexy
- ☐ not-ischaemic cardiomyopathy
- ☐ Valvular heart defect / disease
- ☐ Diabetes mellitus
- ☐ Chronic-obstructive pulmonary disease
- ☐ Chronic liver disease, cirrhosis
- ☐ Nicotine abuse
- ☐ Alcohol abuse
- ☐ Obesity (BMI > 30)

☐ Other: ____

____

____

☐ Not known

22. Had he deceased regularly taken one or more of the following drugs?

- ☐ Nitrate
- ☐ Beta blocker
- ☐ Calcium-Antagonist
- ☐ Antiarrhythmic (other than beta blocker)
- ☐ Digitalis
- ☐ Diuretic
- ☐ Inhibitor of platelet aggregation
- ☐ Anticoagulant
- ☐ ACE-Inhibitor
- ☐ Lipid lowering drug
- ☐ Insulin
- ☐ Oral Antidiabetic

☐ Other: ____

____

____

☐ Not known

Thank you for filling the physician's questionnaire!
